# Characteristics of chronic non-specific musculoskeletal pain in children and adolescents attending a rheumatology outpatients clinic: a cross-sectional study

**DOI:** 10.1186/1546-0096-9-3

**Published:** 2011-01-19

**Authors:** Peter O'Sullivan, Darren Beales, Lynn Jensen, Kevin Murray, Tenielle Myers

**Affiliations:** 1 School of Physiotherapy and Curtin Health Innovation Research Institute, Curtin University of Technology, GPO Box U1987 Perth, 6845, Western Australia; 2 Physiotherapy Department, Princess Margaret Hospital, GPO Box D184, 6840, Perth Western Australia; 3 Rheumatology Department, Princess Margaret Hospital, GPO Box D184, 6840, Perth Western Australia

## Abstract

**Background:**

Chronic non-specific musculoskeletal pain (CNSMSP) may develop in childhood and adolescence, leading to disability and reduced quality of life that continues into adulthood. The purpose of the study was to build a biopsychosocial profile of children and adolescents with CNSMSP.

**Methods:**

CNSMSP subjects (n = 30, 18 females, age 7-18) were compared with age matched pain free controls across a number of biopsychosocial domains.

**Results:**

In the psychosocial domain CNSMSP subjects had increased levels of anxiety and depression, and had more somatic pain complaints. In the lifestyle domain CNSMSP subjects had lower physical activity levels, but no difference in television or computer use compared to pain free subjects. Physically, CNSMSP subjects tended to sit with a more slumped spinal posture, had reduced back muscle endurance, increased presence of joint hypermobility and poorer gross motor skills.

**Conclusion:**

These findings support the notion that CNSMSP is a multidimensional biopsychosocial disorder. Further research is needed to increase understanding of how the psychosocial, lifestyle and physical factors develop and interact in CNSMSP.

## Background

Chronic non-specific musculoskeletal pain (CNSMSP) is common in adolescents and adults, and the prevalence appears to be increasing [1,2]. Often a specific, identifiable pathoanatomical basis for symptoms cannot be found, resulting in non-specific diagnoses based on the location of symptoms. Subjects with CNSMSP have been reported to have relatively high levels of health care utilisation [3], and the presence of CNSMSP negatively impacts physical function, psychological profiles, social functioning and family life [4-8]. Furthermore, CNSMSP can be persistent in a high proportion of children and adolescents [9,10] and may be a strong predictor of CNSMSP in adulthood [11], a trend observed in adolescents with chronic back pain [12-14].

It is generally acknowledged that CNSMSP disorders have a multidimensional biopsychosocial basis [15-19], where these disorders are perpetuated by physical, lifestyle and psychosocial factors that interact to create a vicious cycle of pain [20]. A multidisciplinary treatment approach addressing multiple factors has been shown to have some efficacy in the management of 6-21 year olds with CNSMSP [21]. However, consensus on the classification of CNSMSP disorders according to the presence of factors from multiple dimensions is not always forthcoming [22]. Greater understanding of the factors underlying CNSMSP disorders is needed to assist clinicians in classifying these subjects, and to help guide decision making processes during management.

To date few studies have investigated children and adolescents with CNSMSP from a detailed biopsychosocial perspective, leaving a diagnostic and management vacuum. Therefore the purpose of this study was to investigate children and adolescents from a biopsychosocial perspective, presenting with CNSMSP to a rheumatology outpatients clinic at a children's hospital. This included assessment of various psychosocial factors, known to be risk factors for the development of CNSMSP [11,23]. Additionally, lifestyle factors of physical activity and computer and television use were assessed, as they could potentially contribute to CNSMSP, though their presence in these types of disorders show variable representation in the literature [11,23,24]. From the physical domain joint hypermobility was investigated, for which there is also variable support in the literature for having a role in CNSMSP [11,25]. Other physical factors of sagittal sitting posture, and back muscle endurance (BME) were also investigated, as they have commonly been investigated with musculoskeletal pain disorders but not so commonly in CNSMSP. The results of this study could provide direct insight into the presence of specific biopsychosocial factors in subjects with CNSMSP.

## Methods

### Subjects

Thirty subjects (mean age 12.7 years, range 7-18, 18 females) with a diagnosis of CNSMSP were recruited for this cross-sectional study. The clinical definition of CNSMSP used was: pain present for more than three days per week on average for greater than three months usually associated with interference with or modification of normal function. This diagnosis was made by a paediatric rheumatologist who had clinically and radiologically ruled out specific causes of musculoskeletal symptoms, including rheumatologic, neurologic or orthopaedic disorders. Of this group, 86% reported lumbar spine pain, with 36% of those having concurrent thoracic pain, 40% concurrent cervical pain and 50% concurrent lower limb pain. Thirteen percent reported lower limb pain only. Twenty three percent reported upper limb pain occurring concurrently with other pain sites. Other characteristic included high levels of pain in the last week, moderate disability, significant history of school absenteeism and moderate levels of fear avoidance (Table 1).

**Table 1:** Characteristics of the CNSMSP subjects.

Duration of symptoms	17.3 months (3-26)
Pain score over the previous week [80]	5.64/10 (2-10)
Average days off school in the last year	13.4 days (1-160)
Oswestry Disability Questionnaire [81]	27.3% (6-43)
Tampa Scale of Kinesiophobia [82]	37/68 (29-45)

(mean (range))

Thirty pain free control subjects, matched for age (mean age 13.0 years, range 7-18) and sex, were also recruited. The study was approved by the Princess Margaret Hospital for Children Scientific and Ethics Committee. Written consent was obtained from all parents and the children/adolescents assented to participate.

### Psychosocial Factors Assessment

Psychosocial factors were assessed with the Child Behaviour Checklist [26]. This questionnaire measures eight scales of behaviour: somatic complaints, withdrawn, anxious/depressed, social problems, thought problems, attention problems, delinquent behaviour and aggressive behaviour. It is a valid and reliable tool, frequently used to assess a spectrum of psychosocial behaviours in children and adolescents [27,28].

### Lifestyle Factors Assessment

Lifestyle factors during the previous month were assessed with the Youth Activity Questionnaire [29]. This is a self-report questionnaire chosen as it is specifically designed for the age group participating in this study, with specific questions related to the frequency and duration of physical activity, computer use and television viewing. Frequency was determined on the following scales: 0 = never, 1 = once a month, 2 = once a week, 3 = 2-3 times a week, and 4 = daily. Duration was determined by: 0 = < 30 minutes, 1 = 30-60 minutes, 2 = 1-2 hours, 3 = 2-5 hours, and 4 = >5 hours.

### Spinal Postures Assessment

**Figure 1:**
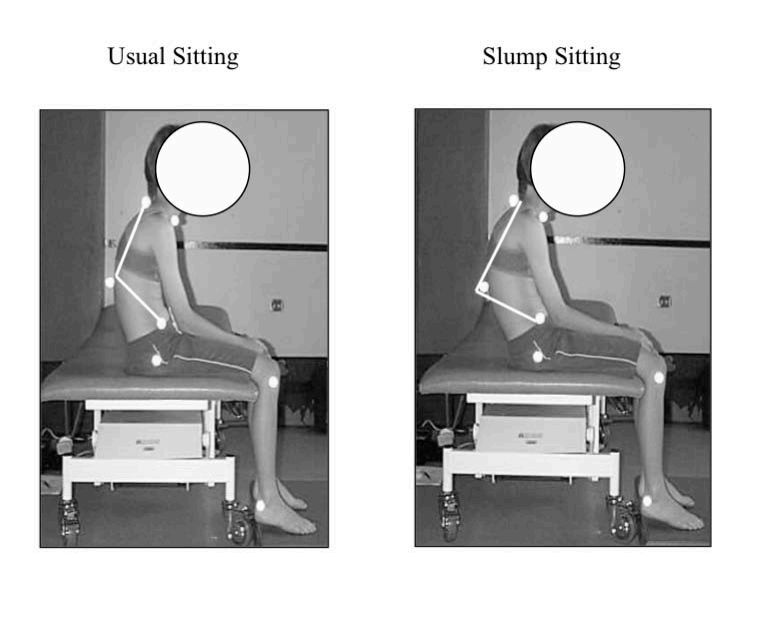
**Thoracolumbar flexion in usual and slump sitting for a subject with CNSMSP **. The difference between these two positions was less in the CNSMSP subjects than the control subjects, suggesting the CNSMSP subjects habitually positioned themselves closer to end range trunk flexion when sitting.

**Figure 2:**
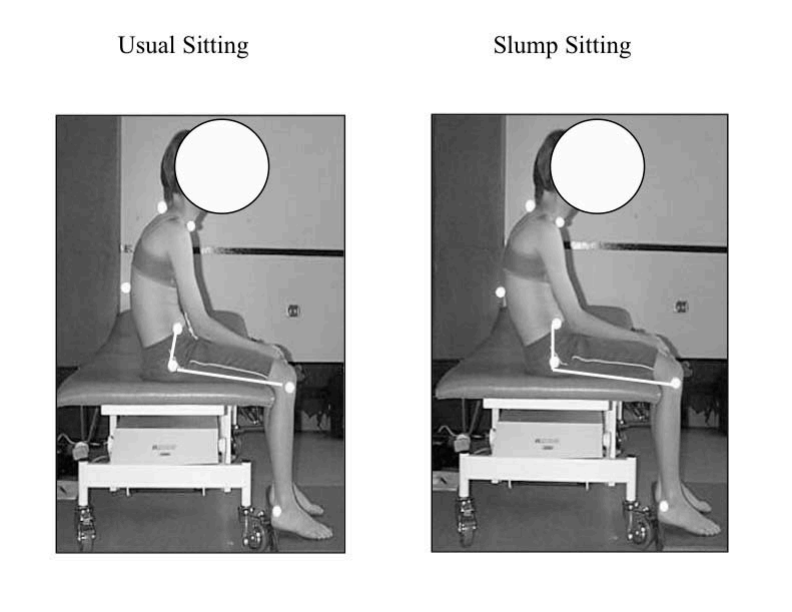
**Pelvic tilt in usual and slump sitting for a CNSMSP subject **. There was a smaller difference between these positions in the CNSMSP group compared to the pain free subjects, suggesting CNSMSP usually sit with their pelvis closer to end range of posterior tilt.

**Figure 3:**
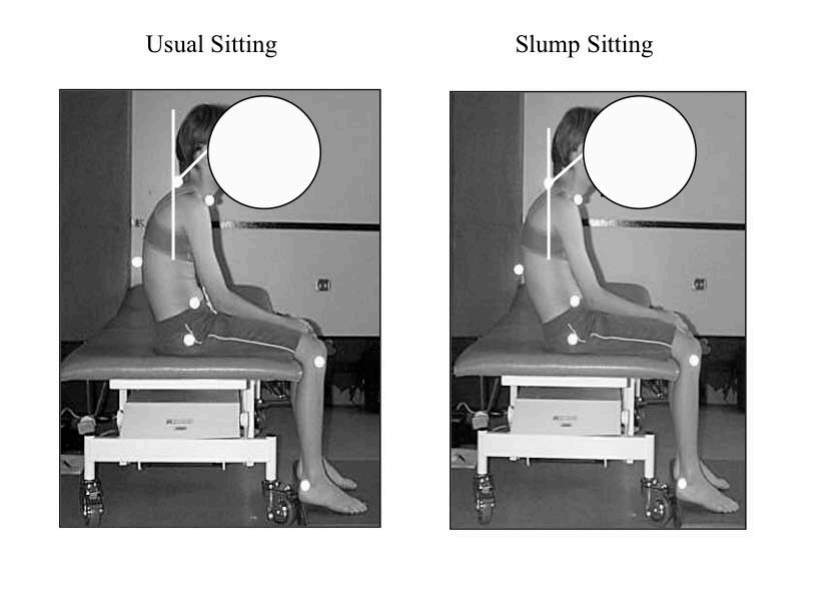
**Cervical flexion during usual and slump sitting for a CNSMSP subject **. CNSMSP subjects sat with more cervical flexion in usual sitting.

Spinal posture was assessed in two positions: (i) 'usual' sitting, and (ii) 'slump' sitting defined as a maximally relaxed spinal posture generally associated with posterior pelvic rotation and trunk flexion. Reflective markers were placed on the spinous processes of C7 and T12, the right anterior superior iliac spine, right greater trochanter and the right lateral condyle of the femur. A digital camera (Sony DSC P72) placed 200 cm from the right greater trochanter in sitting was used to capture photographs of sagittal postural alignment. The images were downloaded to a computer running Scion Image, a computer program that measures angles between marked positions on digital images. The following angles were measured:

• thoracolumbar flexion in usual and slump sitting (angle between a line from C7 to T12, and a line between T12 and the greater trochanter) (Figure 1)

• pelvic tilt (angle between a line from the anterior superior iliac spine to the greater trochanter, and a line from the greater trochanter to the lateral condyle of the femur) (Figure 2)

• cervical flexion in sitting (angle between a vertical line through C7, and a line between C7 and the external auditory meatus) (Figure 3)

• head tilt in sitting (angle between a vertical line through the external auditory meatus, and a line between the external auditory meatus and the outer canthus).

The difference between usual and slump sitting for each measure was also calculated. Two-dimensional computer analysis of lateral photographs has been shown to be a reliable and valid method for evaluating sagittal lumbar and cervical spinal postures [30-32].

### Back Muscle Endurance Assessment

This was measured using the Biering-Sorensen test [33]. The subjects were positioned in prone with their pelvis and thighs stabilised and their trunk unsupported over a plinth. The subjects were asked to hold their trunk parallel to the floor for as long as possible. Time was measured using a stopwatch from the moment the subject achieved a horizontal position until the trunk deviated 15° from the horizontal. An inclinometer was placed on the spine to measure the angle. Subjects were asked to report any pain during testing. The Biering-Sorensen test is a reliable [34] and valid [33] measure of back muscle endurance and has been found to be sensitive in differentiating subjects with spinal pain from healthy controls [35].

### Joint Hypermobility Assessment

Joint hypermobility was measured using the Beighton scale [36]. The subjects were allocated one point for each of the following criteria to give a total score out of 9; metacarpophalangeal extension to 90° or greater, thumb opposition to forearm, elbow hyperextension of 10° or greater, knee hyperextension of 10° or greater, and forward bending in standing with knees straight with hands flat on floor. This has been shown to be a reliable procedure [36].

### Gross Motor Skills

Five tasks comprising the gross motor skill component of the McCarron Assessment of Neuromuscular Development (MAND) [37] were assessed; finger-nose-finger with and without vision (timed test), hand grip strength of both hands, walking heel-toe forward and backwards (steps and movement pattern), single leg stance of both legs with and without vision (timed test) and broad jump (distance and movement pattern). For each task the participant received a raw score, which was then converted to a scaled score according to the subject's age and gender. Individual task scores where added to give a single gross motor score. The MAND is considered a valid and reliable tool in the assessment of gross and fine motor skills [37].

### Data Analysis

Data were coded and analysed for normal distribution. Non-parametric tests were chosen due to an outlier in the control group. Mann-Whitney U tests were used to compare the difference between the pain group and control group for each of the variables with α = 0.05.

## Results

### Psychosocial Factors Assessment

Subjects with CNSMSP had statistically significantly more somatic complaints (p < 0.001) and higher levels of anxiety/depression (p = 0.018) compared to the control subjects (Table 2). There were no differences between groups for the other domains on the Child Behaviour Checklist.

**Table 2:** Psychosocial and lifestyle factors scores for both subject groups.

	CNSMSP	Control	p-value
**Child Behaviour Checklist**			
Somatic complaints	4.7 (2.8)	1.2 (1.4)	<0.001*
Anxious/depressed behaviour	5.4 (3.2)	3.5 (3.6)	0.018*
Withdrawn behaviour	2.2 (1.1)	2.0 (1.2)	0.386
Social problems	1.2 (1.2)	1.4 (1.2)	0.528
Thought problems	0.4 (0.7)	0.4 (0.7)	0.801
Attention problems	1.8 (1.7)	1.7 (1.2)	0.760
Delinquent behaviour	1.7 (3.8)	1.2 (1.0)	0.654
Aggressive behaviour	2.7 (1.6)	2.1 (1.4)	0.736
**Youth Activity Questionnaire**			
Physical activity level	1.3(1.29)	2.83(1.26)	0.005*
Computer use	2.33(1.15)	1.8(1.03)	0.186
Television use	2.23(0.97)	1.76(1.0)	0.484

(mean(standard deviation))

### Lifestyle Factors Assessment

Pain subjects participated significantly less in physical activity (p = 0.005) than the control subjects (Table 2). Forty three percent of CNSMSP subjects reported not participating in any physical activity, while 70% of the control group participated in two to five hours per day.

All subjects reported daily computer use and daily television use, with no statistical difference between the groups in relation to the time spent pursuing these activities (Table 2).

### Spinal Postures Assessment

Subjects with CNSMSP sat in more thoracolumbar flexion during usual sitting compared to pain free controls, but had significantly less thoracolumbar flexion than the controls during slump sitting (p = 0.02) (Table 3). The difference between usual and slump sitting was significantly less in the pain group (p = 0.01) (Figure 1, Table 3), indicating that the CNSMSP subjects habitually sat closer to their end range of thoracolumbar flexion.

**Table 3:** Postural angles for CNSMSP subjects and pain free controls.

	CNSMSP	Control	p-value
**Thoracolumbar Posture**			
Usual sitting	119.8° (10.7°)	125.6° (12.5°)	0.06
Slump sitting	115.6° (10.1°)	110.1° (6.8°)	0.02*
Difference	4.2° (8.7°)	15.5° (11.5°)	0.01*
**Pelvic Tilt**			
Usual sitting	85.2° (13.2°)	82.9° (11.9°)	0.795
Slump sitting	85.9° (17.1°)	88.1° (12.1°)	0.57
Difference	-0.7° (8.7°)	-5.2° (11.5°)	0.05*
**Cervical Flexion**			
Usual sitting	123.5° (11.1°)	132.6° (7.9°)	0.007*
Slump sitting	102.9° (14.5°)	105.5° (13.1°)	0.286
Difference	20.6° (14.5°)	27.1° (10.6°)	0.125
**Head Tilt**			
Usual sitting	94.5° (12.8°)	102.4° (8.4°)	0.107
Slump sitting	84.6° (14.2°)	88.3° (14.2°)	0.374
Difference	9.9° (12.1°)	14.1° (11.6°)	0.511

(mean(standard deviation), negative values for pelvic tilt indicate backwards pelvic tilt)

Pelvic tilt was no different between groups in either usual or slump sitting. However the difference between pelvic tilt in usual and slump sitting was significantly smaller (p = 0.05) in the pain group (Figure 2, Table 3), indicating that the CNSMSP subjects moved through a smaller range of pelvic tilt between these two positions.

Cervical flexion was significantly greater (p = 0.007) in CNSMSP subjects when sitting in their usual posture (Figure 3, Table 3). There was no difference between groups for this measure in slump sitting or with the difference between usual and slump sitting.

No differences between groups were observed in head tilt for usual or slump sitting or the differences between these postures (Table 3).

### Back Muscle Endurance Assessment

Mean (standard deviation) back muscle endurance in CNSMSP subjects at 12.4 (10.2) seconds was significantly less (p < 0.001) than 57.9 (62.0) seconds in the control subjects. Three participants reported discomfort during the test, so the data were re-analysed with these subjects removed to avoid the presence of pain as a confounding factor for the test. The difference between the groups was still significant (p < 0.005).

### Joint Hypermobility Assessment

Subjects with CNSMSP had, on average, hypermobility in 5.0 (2.5) joints. This was significantly more (p = 0.046) than the control subjects with on average 3.7 hypermobile joints.

### Gross Motor Skills

The gross motor component of the MAND was significantly less (p = 0.028) in the pain group (mean 48.2, standard deviation 12.8) than the control group (mean 55.1, standard deviation 5.6).

## Discussion

This study documents the characteristics of a group of children/adolescents with CNSMSP who have high levels of pain and disability, significant school absenteeism and moderate levels of fear avoidance (Table 1). This is clearly a disabled group attending tertiary referral from their general medical practitioners due to the impact of their pain. The majority had low back pain. These subjects displayed clear differences in psychosocial, lifestyle and physical profiles compared to matched pain free controls. This supports the assumption of a multifactorial biopsychosocial presentation for CNSMSP. The clinical diagnosis of CNSMSP in this study was made where structural and specific rheumatic disorders had been clinically and radiologically excluded. Furthermore, the CNSMSP subjects in this study are from a wide age range. Despite these limitations, the specific findings of the study support this disorder is associated with impairments across a range of different domains. These findings refute previous reports that musculoskeletal pain in children and adolescents is not associated with disability and significant impairment [38,39].

### Psychosocial Factors

It is widely known that negative psychosocial factors are associated with a large range of chronic musculoskeletal disorders. Consistent with this is the finding of higher levels of anxiety/depression in CNSMSP subjects. This matches reports that depression is a risk factor for schoolchildren who develop CNSMSP [11], and anxiety and depression have been described in other groups of children/adolescents with CNSMSP [7,40]. It is not known what the relationship between the increased depression and anxiety levels have to the pain disorders due to the cross sectional nature of the study. The presence of pain may result in increased anxiety and depression, but conversely altered mood is known to influence pain modulation [15]. In either case these factors are likely to interact, reinforcing the vicious cycle nature of chronic pain [41].

In contrast to other findings, the present study did not detect differences in other behaviours such as aggressive, delinquent or withdrawn behaviour or social problems between pain subjects and pain free controls (Table 2), which contrasts to previously reported associations between negative behaviour traits and CNSMSP in children [23]. This may indicate that sub-groups of subjects with CNSMSP exist with different psychosocial profiles or reflect the small sample size in this study. Less layered approaches to psychosocial profiles, such as general description of high or low presence of these factors [42], may not be sensitive enough for the purpose of sub-grouping these subjects. Further research is warranted to investigate this notion.

Though not strictly a psychosocial factor, data from the Child Behaviour Checklist did reflect increased reports of comorbid pain complaints in the CNSMSP subjects. From a clinical perspective, in none of these patients was another evident diagnosis or direct cause of these other somatic complaints identified. This particular relationship has been reported previously in children [11,23] and in more general terms the presence of co-morbidities to more specific chronic musculoskeletal conditions has been documented [24,43-48]. It is unknown if co-morbid conditions exist as independent clinical entities [49], or if they are related by a common underlying pathological basis [49-52]. Further research is needed to investigate the relationship between chronic musculoskeletal conditions and co-morbidities, and to investigate for the existence of different clusters of somatic co-morbidities.

### Lifestyle Factors

The CNSMSP subjects in this study reported lower levels of physical activity than pain free controls. The role of physical activity in CNSMSP is likely to be complex though. Increased, not decreased, physical activity levels have previously been reported as a risk factor for the development of musculoskeletal pain in school children [23,53]. This may be consistent of a 'U-shape' relationship between physical activity levels and CNSMSP, as described in chronic low back pain where either too little or too much physical activity may be a risk factor for the development of symptoms [54]. Further more, other studies have found no relationship between physical activity levels and CNSMSP [11], perhaps indicating the exact nature of the physical activity undertaken may be important. Additionally, it remains to be determined if reduced exercise participation is a secondary effect of pain or a significant factor in the pathogenesis of CNSMSP. Further research is warranted investigating to role of physical activity levels in CNSMSP.

Similar to a previous report [24], television and computer use were not found to be significantly different in CNSMSP subjects. Interestingly though television use and computer use have been reported as factors in more localised childhood/adolescent musculoskeletal pain disorders [55-58]. Perhaps the power of this study was not sufficient to detect differences in these variables. If the pain subjects were less physically active, but not spending more time on computers or watching television, it would be interesting to determine what the pain subjects were doing in this extra time.

### Spinal Postures

While relationships between sitting and CNSMSP do not appear to have been examined in the literature, sitting has been identified as a risk factor for localised musculoskeletal spinal pain [59-61]. The results of this study appear to be one of the first to document alterations in sitting posture in subjects with CNSMSP. The cumulative result of the specific, identified features of the sitting posture in CNSMSP subjects was that they tended to sit more slumped, closer to end range of spinal flexion (Figure 1, Figure 2, Figure 3). This could potentially reduce muscular support [30,62-65] and may increase strain on passive spinal structures [66], thus providing a physical mechanism for symptom development and provocation. Even though the time spent by the pain subjects in this study watching television or using a computer was not significantly different from pain free subjects, these activities may be problematic due to the postures adopted during these tasks. Future studies should endeavor to relate sitting posture to specific sitting related activities that result in increased symptoms to improve understanding of the relationships between these factors.

In usual sitting, the CNSMSP subjects had greater cervical flexion than the pain free subjects. This contrasts to adolescents with neck/shoulder pain [67], perhaps pointing to the existence of subgroups of subjects who adopt different cervical postures. Though the tendency on average was for the CNSMSP subjects to sit more slumped during usual sitting, individual variation is apparent from some of the standard deviation in the postural angles data (Table 3), which may mean that in future studies subgroups of sitting posture can be identified as has been the case in a group of adolescents with non-specific chronic low back pain [68,69].

Furthermore, with increased cervical flexion in usual sitting it might be expected that CNSMSP subjects would tilt their heads further back further to increase their visual field. The lack of differences between the pain and pain free subjects for head tilt though suggest that this was not the case, potentially meaning that the pain subjects would tend to gaze at the ground rather than directly forwards. This adaptation might be consistent with/related to the negative psychosocial factors, supporting the concept of mind-body relationships [70,71], where slump sitting has been found to be associated with negative emotions and poorer motivation [72-74]. Further study of this phenomenon is warranted.

### Back Muscle Endurance

Back muscle endurance was significantly less in the subjects with CNSMSP. It does not appear to have been specifically investigated in children or adolescents with CNSMSP previously. There are mixed findings with this variable in those age groups with low back pain [75], though a recent study has reported reduced back muscle endurance in adolescents with non-specific chronic low back pain [68].

Though pain during testing did not appear to be a factor in the finding of reduced back muscle endurance, pain in general and/or reduced physical activity levels could be responsible for this finding. It could also be linked to poor motivation and negative psychosocial traits. Reduced back muscle endurance could then potentially contribute to the adoption of more slumped postures. However, given the possibility of muscle relaxation in slump postures [30,62-65], conversely reduced endurance could be a secondary effect of slumping. Certainly reduced back muscle endurance has been associated with slump sitting posture in industrial workers with flexion related BP [35]. Further research is warranted to investigate these relationships in CNSMSP.

### Joint Hypermobility

While some previous studies have suggested a relationship between joint hypermobility and CNSMSP [76,77], others have not [11,53,78]. The results of this study show that there is at least a subgroup of subjects with CNSMSP who have on average more hypermobile joints than pain free subjects. Certainly not all subjects with joint hypermobility have widespread CNSMSP though, with 14% of 107 hypermobile subjects reporting diffuse musculoskeletal pain in a previous study [25]. Hypermobility has been variably linked to delayed gross and fine motor performance [77], which was consistent with reduced gross motor skills observed in the CNSMSP group of this study.

### Clinical implications

Despite the limitation of a cross-sectional design, small sample size and non-blinding of the investigators, the findings support that a subgroup of children and adolescents exist with CNSMSP who attended a Rheumatology Outpatients department. The impact of this is significant, disabling and is associated with significant impairments across multiple domains. This provides an interesting contrast to a group of adolescent with non-specific chronic LBP, who had considerable pain and impact, but did not demonstrate difference in the psychosocial and lifestyle domains seen in the present study [68]. The comparison of that study to the present study is consistent with findings in adults of a sub-group of subjects with higher levels of psychosocial involvement and disability in contrast to a sub-group with lower pain, psychosocial involvement and disability [79].

This finding refutes previous suggestions that these disorders are rarely disabling [38,39]. Furthermore it supports calls to investigate and manage these disorders from a biopsychosocial perspective [15,17,18]. Clearly further research is required to investigate the evolution of these disorders in order to further understand the underlying mechanisms associated with them. However these disorders should be considered as serious due to their disabling nature, psychosocial impact, and associated activity avoidance and high levels of school absenteeism. There is evidence that these disorders have a tendency to predict future adult chronic musculoskeletal pain [11] highlighting that early intervention is critical.

## Conclusion

This study has identified clinical features of a specific group of subjects with CNSMSP. The findings support an underlying multifactorial basis as psychosocial, lifestyle and physical factors were all found to play a role in the pain subjects. Further research is required, with larger numbers of subjects, to expand the profile of these subjects and to increase our understanding of how multiple domain factors may interact. The results suggest that treatment should follow a cognitive-functional approach to [17] address the multi-dimensional, biopsychosocial nature of the disorder. Further investigations into the evolution and management of these disorders are required.

## Abbreviations

CNSMSP: chronic non-specific musculoskeletal pain; MAND: McCarron Assessment of Neuromuscular Development

## Competing interests

The authors declare that they have no competing interests.

## Authors' contributions

Substantial contributions to conception and design: PO'S, LJ, KM, TM. Substantial contributions to acquisition of data: PO'S, LJ, KM, TM. Substantial contributions to analysis and interpretation: PO'S, DB, LJ, KM, TM. Involved in drafting the manuscript or revising it critically for important intellectual content: PO'S, DB, LJ, KM, TM. Have given final approval of the version to be published: PO'S, DB, LJ, KM, TM.
